# Vaginal myomectomy for a large intrastromal cervical fibroid: a case report

**DOI:** 10.1093/jscr/rjae797

**Published:** 2024-12-17

**Authors:** Yousef Alebrahim, Ee Thong Lim, Theofanis Manias, Nikolaos Tsampras

**Affiliations:** Department of Reproductive Medicine, Manchester University NHS Foundation Trust, Oxford Rd, Manchester, WL, United Kingdom; Department of Gynaecology, Manchester University NHS Foundation Trust, Oxford Rd, Manchester, WL, United Kingdom; Department of Gynaecology, Manchester University NHS Foundation Trust, Oxford Rd, Manchester, WL, United Kingdom; Developmental Biology and Medicine, School of Medical Sciences, The University of Manchester, Oxford Rd, Manchester, PL, United Kingdom

**Keywords:** vaginal surgery, myomectomy, cervical fibroid, leiomyoma

## Abstract

Fibroids affect up to two-thirds of women of reproductive age and can lead to significant symptoms, including abnormal uterine bleeding and reproductive dysfunction. Cervical fibroids are rare, with an incidence of only 0.6%. Currently, there is no consensus on the optimal surgical approach for management of cervical fibroids. Pedunculated extracervical fibroids are routinely removed vaginally. However, intrastromal (intracervical) fibroids often require a laparoscopic or laparotomic approach. We report a case of vaginal myomectomy for the management of a large cervical fibroid in a 43-year-old patient seeking fertility treatment. We discuss the feasibility of this surgical approach and its potential for enhanced recovery.

## Introduction

Fibroids, or leiomyomas, are the most common benign tumours of the uterus, affecting up to 60% of women of reproductive age [1]. Cervical fibroids are a rare subtype, with an incidence of only 0.6% [2]. Approximately one in three women with fibroids experience symptoms such as abnormal bleeding and pelvic pressure. Additionally, women with fibroids are at an increased risk of subfertility, recurrent miscarriage and obstetric complications [1].

Whilst surgery is the standard treatment of cervical fibroids, there is currently no consensus on the optimal surgical approach. Fertility-sparing surgery for intracervical fibroids can be particularly challenging due to the high risk of haemorrhage and potential injuries to the adjacent organs that are often dislocated and contiguous to the cervical leiomyoma [2, 3].

We present a case of a vaginal myomectomy for a large intrastromal cervical fibroid in a patient undergoing assisted conception treatment. We discuss the clinical implications of cervical fibroids and the feasibility of this surgical approach.

## Case report

A 43-year-old female presented to the emergency department with heavy vaginal bleeding during an in vitro fertilisation (IVF) cycle. She had a history of two surgical terminations of pregnancy and no other significant medical history.

On assessment, the patient was haemodynamically stable. Abdominal examination was unremarkable. Vaginal examination revealed a bulging cervix containing a large mass, with the external cervical os displaced posteriorly.

The patient was admitted due to ongoing bleeding and symptomatic anaemia. She received tranexamic acid and a single unit of packed red blood cells. Pelvic ultrasound revealed a heterogenous mass within the cervical canal measuring 6 cm suggestive of a fibroid. The patient was discharged after a 5-day inpatient stay. The IVF cycle was cancelled due to suboptimal response in keeping with low ovarian reserve. Subsequently, a 3-month course of leuprorelin acetate injections was commenced.

**Figure 1:**
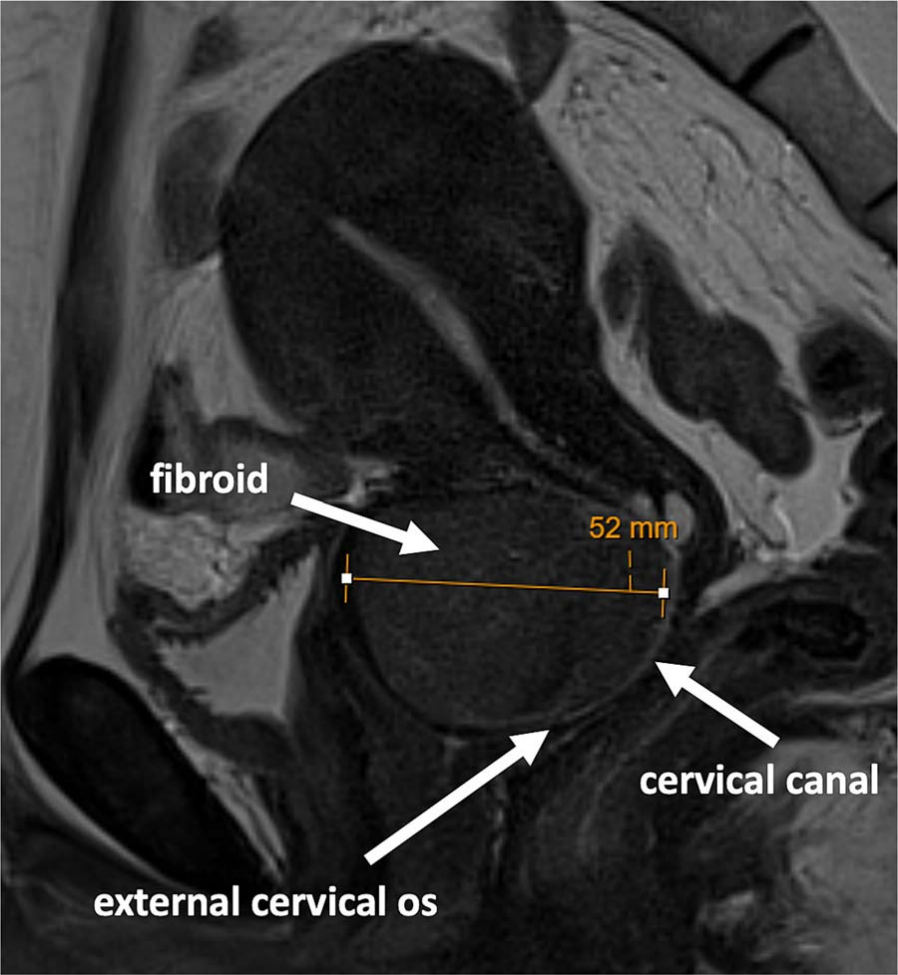
MRI scan showing large fibroid within the anterior cervical stroma with compression of the cervical canal.

An outpatient magnetic resonance imaging (MRI) scan demonstrated a well-defined homogenous mass within the anterior cervix measuring 5.2 cm in maximum dimension (Fig. 1).

The rest of the pelvic viscera appeared normal.

**Figure 2:**
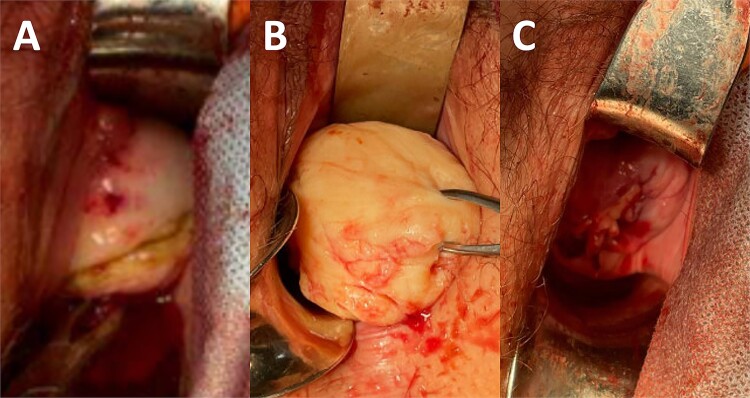
(A) Incision in the anterior lip of the cervix. (B) Enucleated cervical fibroid. (C) Closure of incision at the end of the procedure.

An elective admission was arranged for vaginal myomectomy. The procedure was undertaken with the patient in Lloyd-Davies position. Perioperative antibiotics were administered. The bladder was decompressed by an indwelling catheter. Twenty units of vasopressin were diluted with 80 mL of normal saline solution, and 30 mL of the diluted solution was injected into the fibroid pseudocapsule. An oblique incision was made in the anterior lip of the cervix, aiming to prevent an extension to the bladder. The fibroid was enucleated using blunt and sharp dissection and retrieved as a single specimen (Fig. 2).

The remaining cavity and cervical incision were closed using a single continuous suture with polyglactin. A diagnostic hysteroscopy demonstrated an intact cervical canal, regular uterine cavity, and normal endometrium. Hyaluronic acid gel was instilled into the cervical canal for prevention of intracervical adhesions. Blood loss during the procedure was minimal (<50 mL). She recovered well and was discharged later that day.

**Figure 3:**
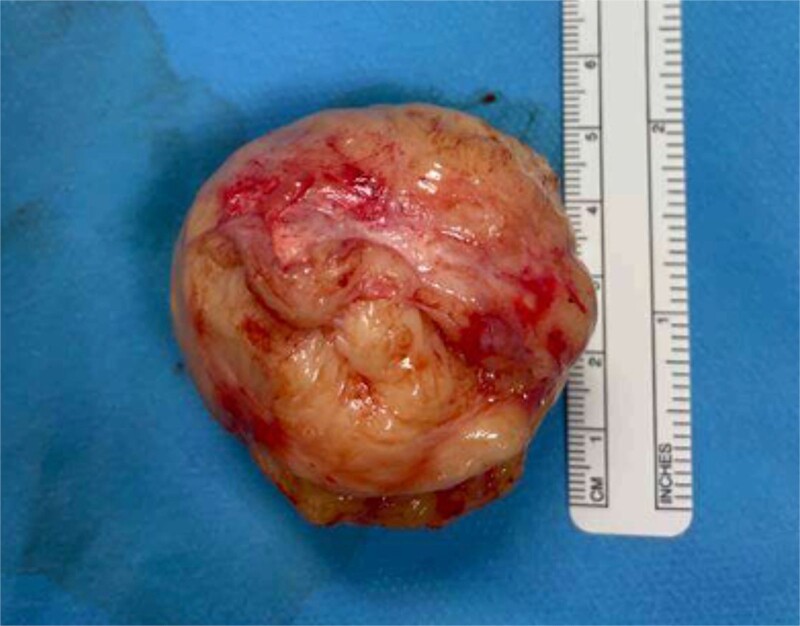
Benign leiomyoma retrieved following vaginal myomectomy.

Histological examination of the 5.0 × 4.7 × 4.3 cm mass confirmed a benign leiomyoma (Fig. 3).

At 4-month follow-up, the patient had made a full recovery and reported significant improvement of her periods. She was considering further fertility treatment with possible use of donor eggs.

## Discussion

Management of cervical fibroids presents a challenge due to their rare incidence. The choice of treatment is crucial for preserving fertility in women of reproductive age. Surgical intervention is often necessary to improve bleeding symptoms and reproductive outcomes. Traditional approaches with laparoscopy and laparotomy are associated with increased risk of morbidity and recovery times [2]. The vaginal approach presented in this case demonstrates a minimally invasive technique with shorter operative time, minimal blood loss, and enhanced recovery. Vaginal myomectomy with colpotomy has been previously described for removal of fibroids from the uterine body [4]. However, this has not been reported for management of intrastromal cervical fibroids without hysterectomy.

Whilst medical options do not provide permanent treatment for fibroids, the use of gonadotropin releasing hormone analogues pre-operatively are effective for reduction of fibroid size and intraoperative bleeding [5]. Injection of vasopressin into the fibroid capsule intraoperatively is an effective haemostatic measure, especially as tourniquet application is not possible [6].

Women with fibroids are at risk of subfertility and miscarriage, with such effects dependent on the fibroid size and location [3]. Cervical fibroids can cause significant distortion of the cervical anatomy including obliteration of the cervical canal. As a result, this can impair transport of spermatozoa and successful cannulation of the cervix during procedures such as tubal patency tests and embryo transfer. Furthermore, fibroids can alter uterine contractility and blood supply significantly [1]. Myomectomy is effective for improving menorrhagia and rates of successful embryo implantation [1, 5].

In this case, we present a favourable outcome utilizing a vaginal approach for a large cervical fibroid. The patient made a rapid and uneventful recovery with significant symptom improvement.

## Conclusion

Vaginal myomectomy is a feasible and safe approach for management of cervical fibroids, with reduced risk of morbidity and enhanced patient recovery.
